# Storing high temperature solar thermal energy in shallow depth artificial reservoir for space heating

**DOI:** 10.1038/s41598-022-24003-0

**Published:** 2022-11-15

**Authors:** Xianbiao Bu, Kunqing Jiang, Zhipeng Guo

**Affiliations:** 1 School of Energy Science and Engineering, University of Science and Technology of China, Guangzhou, 510640 China; 2 Guangzhou Institute of Energy Conversion, Chinese Academy of Sciences, Guangzhou, 510640 China

**Keywords:** Geothermal energy, Solar energy

## Abstract

The discontinuous and unstable characteristics of solar energy limit its application in the space heating field, while aquifer thermal energy storage (ATES), as a seasonal thermal energy storage pattern, is a feasible way of solving these problems faced by solar space heating and however, low temperature ATES must not exceed 25–30 °C while high temperature ATES has low recovery efficiency. Here a novel scheme of storing high temperature solar thermal energy into a shallow depth artificial reservoir (SDAR) is proposed. By innovatively storing thermal energy into rocks rather than aquifer, the recovery efficiency improves from 46% for ATES to 90% for SDAR, and the thermal power increases from 309 kW for deep borehole heat exchanger to 1970 kW for SDAR. SDAR has no special requirement to rock temperature and can thus be created in shallow buried depth rocks, leading not only to a reduction of engineering cost but also an expansion of application scope. To further avoid risk of induced seismicity caused by hydraulic fracturing and reduce cost, the abandoned oil and gas fields and mines can be reused as the artificial reservoir.

## Introduction

The total floor area in China is 644 × 10^8^ m^2^ at present, and its energy demand accounts for about 28% of the total energy use^1,2^. The district heating area in China reached 122.66 × 10^8^ m^2^ by 2020, and 83% of this area was heated by coal-based fuel^3–5^, consuming a lot of energy and causing serious pollutant. Therefore, it is essential to increase the share of renewable energy in the space heating fields in order to reduce greenhouse gas emission.

Geothermal and solar energy resources are abundant in China^6,7^, and they should be the primary choice for renewable energy space heating. However, the total area of geothermal heating (cooling) only reached 13.9 × 10^8^ m^2^ by 2020 due to the dependence on the hydrothermal resources and their unbalanced space distribution^8,9^. While for solar space heating, the area was only 16.50 × 10^6^ m^2^ by 2020 due to the discontinuous and unstable energy characteristics^10^. Seasonal thermal energy storage (STES) is a feasible way of solving the problems faced by solar space heating, among them underground thermal energy storage (UTES) is considered to be the most promising storage technology^11–13^. Aquifer thermal energy storage (ATES), especially low temperature ATES (LT-ATES), is the most suitable UTES system, and about 3000 LT-ATES projects were in operation in Europe by the end of 2017^14^. The temperature of the injected water in LT-ATES system is not allowed to be above 25–30 °C in most countries^15,16^, and it is thus inappropriate to store solar thermal energy with high temperature. Recently, high temperature aquifer thermal energy storage (HT-ATES) has received more and more attentions due to higher storage temperature and larger storage capacities and however, low thermal recovery efficiency due to the effects of free convection caused by the density difference limits its application and this is an urgent and important task that need to be solved at present^17–20^.

The hydrothermal resources are not rich in most regions in China, and therefore the thermal energy and underground space have not been exploited in these regions. Enhanced geothermal system (EGS) is a feasible way to extract geothermal energy stored in the rocks without rich hydrothermal resources and however, the conventional EGS generally requires high rock temperature with a shallow buried depth in order to acquire more thermal energy and reduce the project expenses, limiting the scope of its use. If more thermal energy can still be acquired even if EGS project is built with a shallow depth and a normal rock temperature, it will reduce engineering cost and expand application scope. On this basis, a novel scheme of high temperature solar thermal energy storage into a shallow depth artificial reservoir (HTSTESSDAR) created in the rocks without rich hydrothermal resources is proposed^21–23^. During the non-heating season, the high temperature solar thermal energy is stored into the shallow depth artificial reservoir (SDAR), leading to an increase in the rock temperature inside the artificial reservoir. During the heating season, more thermal energy can be extracted from the artificial reservoir for space heating due to having high rock temperature caused by the thermal energy storage, at the same time the temperature of rocks inside the artificial reservoir also decreases due to the thermal energy extraction. Similarly, more solar thermal energy can be stored into the artificial reservoir during the non-heating season due to having low rock temperature caused by the thermal energy extraction. The thermal transfer medium (water) flows in the fractures created in the artificial reservoir and exchanges heat with the surrounding rocks, thus realizing thermal energy storage mainly in the rocks rather than aquifer (water). Storing thermal energy into the rocks is mainly by heat conduction, which can restrain the natural conversion causing a low thermal recovery efficiency in HT-ATES. It is thus expected that the thermal recovery efficiency can be greatly improved by storing thermal energy in the rocks instead of water. The shallow depth artificial reservoir (also called shallow depth enhanced geothermal system, SDEGS for short) has no special requirement to temperature, depth and storage space for water, and thus leading not only to a considerable reduction of project cost but also a great expansion in the application scope. Fortunately, consolidated sandstone, limestone and granite resources and so on with the shallow buried depth are found to be widely distributed in northern China. For this reason, SDAR or SDEGS is suitable for storing high temperature thermal energy, and however, the thermal performance especially the thermal recovery efficiency for SDAR needs to be testified further by numerical simulation and experiment as well as theoretical analysis. Therefore, the key point of this study is to evaluate and discuss the performance of thermal energy storage and thermal recovery efficiency for HTSTESSDAR from technological perspective by means of numerical simulation.

## Methods

### Physical model

**Figure 1:**
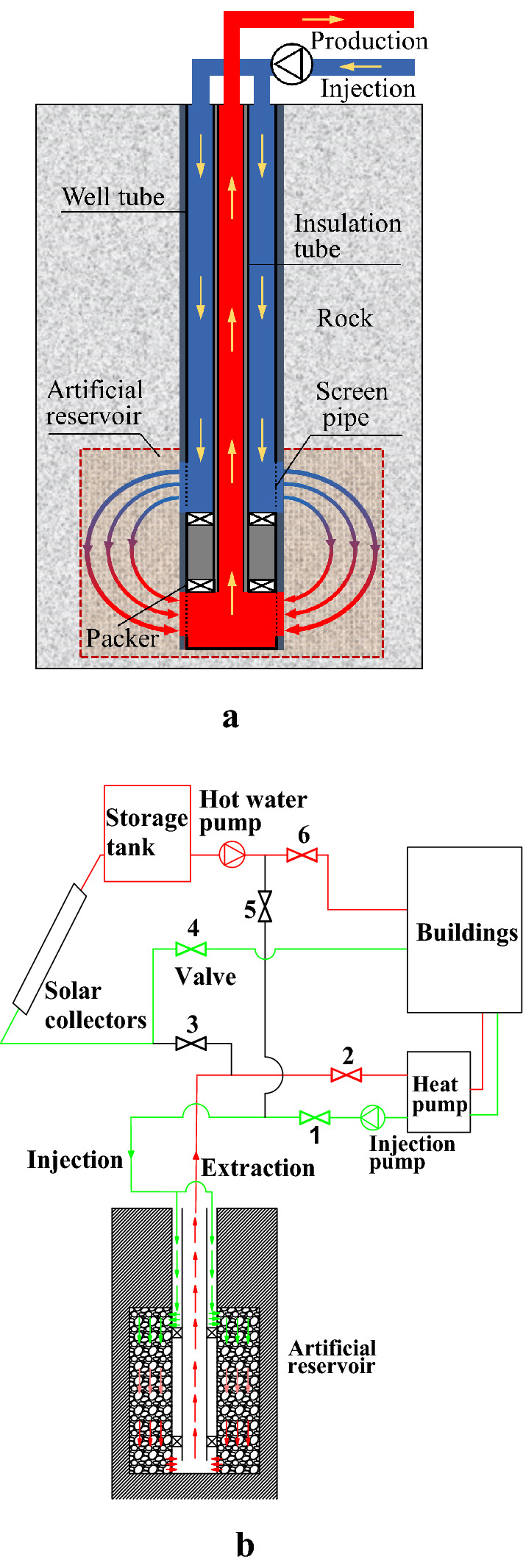
Physical models. (**a**) SDAR system; (**b**) System diagram of HTSTESSDAR.

The physical model of the artificial reservoir system created in the shallow buried depth rocks is shown in Fig. 1a, and it mainly consists of rock, artificial reservoir, insulation tube, well tube, packer, screen pipe, and so on. The artificial reservoir with a height of 130 m and a radius of 30 m is created by the hydraulic fracturing technology, and its depth ranges from 1370 to 1500 m. Two sets of screen pipes are respectively installed at the upper part and the lower part of the artificial reservoir, and two packers are accordingly equipped between two sets screen pipes in order to force the working fluid to flow into the artificial reservoir. The ring-shaped channel between the insulation tube and the well tube is used as the injection channel, and the insulation tube is used as the extraction channel. The circulating water flows into the injection channel and enters into the artificial reservoir through the screen tube, where it exchanges heat with the surrounding rocks and then the heated water or cooled water flows upward along the extraction channel. Tables 1 and 2 list the physical parameters of surrounding rocks, artificial reservoir, well tube and insulation tube.

**Table 1:** Physical parameters of surrounding rocks and artificial reservoir.

Parameter	Artificial reservoir	Surrounding rocks
Density/kg m^−3^	2900	2900
Specific heat/J kg^−1^ K^−1^	705	705
Thermal conductivity/W m^−1^ K^−1^	4.5	4.5
Permeability/m^2^	5 × 10^–15^	1 × 10^–18^
Porosity	0.07	0.01

**Table 2:** Parameters of well tube and insulation tube.

Parameter	Value
Well tube outer diameter/mm	177.8
Well tube thickness/mm	6.91
Insulation tube outer diameter/mm	110
Insulation tube thickness/mm	10
Thermal conductivity of insulation tube/W m^−1^ K^−1^	0.1
Thermal conductivity of well tube/W m^−1^ K^−1^	45

The system diagram of high temperature solar thermal energy storage in shallow depth artificial reservoir (HTSTESSDAR) is shown in Fig. 1b. In Fig. 1b, the evacuated tubular solar collector is used to generate high temperature thermal energy. The high temperature solar thermal energy is stored into the artificial reservoir during the non-heating season, and it is extracted during the heating season for space heating. By the seasonal thermal energy storage, the problems of intermittence and instability of solar energy can be solved. The storage tank just stores short-term heat energy on a diurnal basis in Fig. 1b. During the non-heating season, valves No. 3 and 5 keep open and valves No. 1, 2, 4 and 6 are shut off, and solar thermal energy with a temperature of 90 °C is stored into the artificial reservoir. During the heating season, valves No. 1, 2, 4 and 6 are opened and valves No. 3 and 5 keep closed, and the thermal energy stored in the artificial reservoir is extracted for space heating. In addition, solar energy is also used for the space heating when its temperature meets the requirement. The supply and return water temperatures during the heating season are respectively 40 and 32 °C in the user sides.

To get more thermal energy, the heat pump is used in order to attain a lower injection temperature during the heating season.

### Mathematical model

Based on the conservation of mass, momentum and energy, the mathematical model describing the flow and heat exchange in the well tube, artificial reservoir and insulation tube is given as follows^24,25^:

1. Mathematical model for well tube and artificial reservoir
  Energy equation in the well tube and insulation tube:
  1 $$ \rho_{{\text{f}}} C_{{\text{f}}} A_{{\text{c}}} \frac{\partial T}{{\partial t}} + \rho_{{\text{f}}} C_{{\text{f}}} u_{{\text{f}}} A_{{\text{c}}} \cdot \nabla T - \nabla \cdot \left( {A_{{\text{c}}} \lambda_{{\text{f}}} \nabla T} \right) = Q $$
  The heat transfer item in the insulation tube is expressed as:
  2 $$ Q_{{\text{I}}} = \frac{{T_{{\text{I}}} - T_{{\text{E}}} }}{{R_{{\text{I}}} }} $$
  3 $$ R_{{\text{I}}} = \frac{1}{{2\pi r_{{{\text{II}}}} h_{{{\text{II}}}} }} + \frac{{\ln \left( {r_{{{\text{IO}}}} /r_{{{\text{II}}}} } \right)}}{{2\pi \lambda_{{\text{I}}} }} + \frac{1}{{2\pi r_{{{\text{IO}}}} h_{{{\text{IO}}}} }} $$
  The heat transfer item in the injection channel is given as:
  4 $$ Q_{{\varvec{W}}} = \frac{{T_{{\text{E}}} - T_{{\text{I}}} }}{{R_{{\varvec{I}}} }} + \frac{{T_{{\text{s}}} - T_{{\varvec{I}}} }}{{R_{{\varvec{W}}} }} $$
  5 $$ R_{{\text{W}}} = \frac{1}{{2\pi r_{{{\text{WI}}}} h_{{{\text{WI}}}} }} + \frac{{\ln \left( {r_{{{\text{WO}}}} /r_{{{\text{WI}}}} } \right)}}{{2\pi \lambda_{{\varvec{W}}} }} $$
  The convective heat transfer coefficient between fluid and the tube wall is calculated according to the following formula:
  6 $$ h = \frac{{Nu \cdot \lambda_{{\varvec{f}}} }}{D} $$
  7 $$ Nu = \frac{(f/8) \cdot (Re - 1000) \cdot Pr}{{1 + 12.7\sqrt {f/8} \left( {Pr^{{2{2/3}}} - 1} \right)}} $$
  8 $$ f = \frac{1}{{(1.82\lg Re - 1.64)^{2} }} $$
  The energy conservation for the surrounding rocks around the well section above the artificial reservoir is calculated by:
  9 $$ \rho_{{\text{s}}} C_{{\text{s}}} \frac{{\partial T_{{\text{s}}} }}{\partial t}{ = }\lambda_{{\text{s}}} \frac{1}{r}\frac{\partial }{\partial r}\left( {r\frac{{\partial T_{{\text{s}}} }}{\partial r}} \right) + \lambda_{{\text{s}}} \frac{{\partial^{2} T_{{\text{s}}} }}{{\partial z^{2} }} $$
  The artificial reservoir is assumed to be isotropic and homogeneous and is equivalent to a porous medium having uniform porosity and permeability. The local thermal equilibrium between the porous rock matrix and fluid is considered, and the effective specific heat and thermal conductivity in the artificial reservoir are given as the following.
  10 $$ \left( {\rho C_{{\text{p}}} } \right)_{{\text{e}}} = \phi \left( {\rho_{{\text{f}}} C_{{\text{f}}} } \right) + (1 - \phi )\left( {\rho_{{\text{s}}} C_{{\text{s}}} } \right) $$
  11 $$ \lambda_{{\text{e}}} = \phi \lambda_{{\text{f}}} + (1 - \phi )\lambda_{{\text{s}}} $$
  The energy conservation in the artificial reservoir is given by:
  12 $$ \left( {\rho C_{{\text{p}}} } \right)_{{\text{e}}} \frac{\partial T}{{\partial t}} + \rho_{{\text{f}}} C_{{\text{f}}} q\nabla T - \nabla \cdot \left( {\lambda_{e} \nabla T} \right) = {0} $$
  The mass and momentum conservation equations in the artificial reservoir are calculated according to the following formulas:
  13 $$ \frac{{\partial \left( {\rho_{{\text{f}}} \phi } \right)}}{\partial t} + \nabla \cdot \left( {\rho_{{\text{f}}} q} \right) = {0} $$
  14 $$ q = - \frac{k}{{\mu_{{\text{f}}} }}\nabla \cdot \left( {p + \rho_{{\text{f}}} gz} \right) $$
2. Mathematical model for the evacuated tubular solar collector
  The thermal efficiency for the evacuated tubular solar collector is given below:
  15 $$ TE = 0.721 - 0.89\frac{{T_{m} - T_{{\text{A}}} }}{G} - 0.0199\frac{{\left( {T_{m} - T_{{\text{A}}} } \right)^{2} }}{G} $$
  Solar direct radiation intensity can reach 750 W/m^2^ for 7 h per day during the non-heating season with external average temperature of 21.2 °C, and it is 500 W/m^2^ for 5 h per day during the heating season with external average temperature of 4.6 °C in North China.

### Initial and boundary conditions

The surface temperature is 15 °C and the geothermal gradient is 30 °C/km. The heating duration is 120 days and during this period the heat is extracted from the artificial reservoir with an injection temperature of 10 °C and a volume flow rate of 50 m^3^/h. During the non-heating season with duration of 245 days, the high temperature thermal energy produced from the evacuated tubular solar collector is injected into the artificial reservoir with a temperature of 90 °C and a volume flow rate of 20 m^3^/h. For HTSTESSDAR, the thermal energy storage comes before its extraction.

### Experiment verification

**Figure 2:**
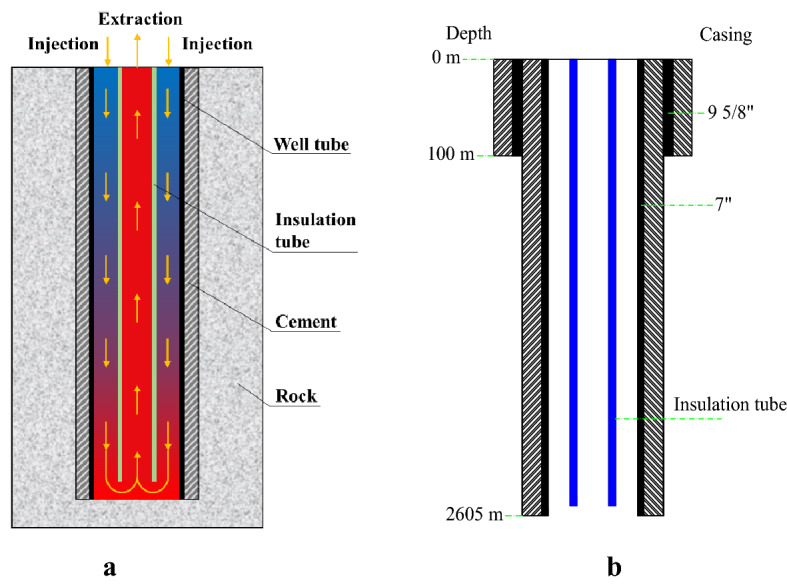
Deep borehole heat exchanger. (**a**) Schematic; (**b**) casing profile.

**Figure 3:**
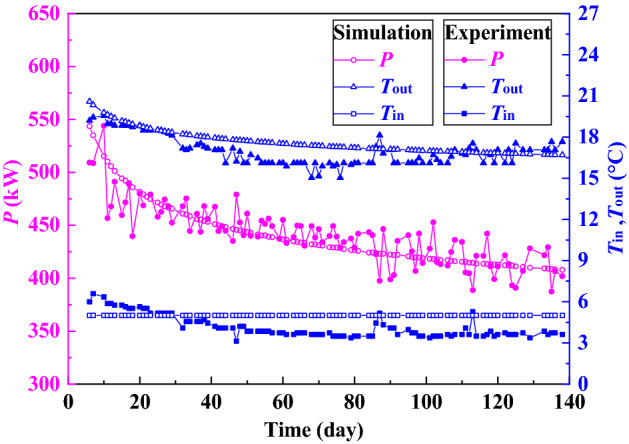
Simulation and experimental results.

The deep borehole heat exchanger (DBHE) was designed and built in 2016 at Qingdao in China, and its schematic and casing profile are shown in Fig. 2. The material of the insulation tube is polypropylene. Other parameters are listed in Table 3. The temperature of injection water was set at around 5 °C and the volume flow rate was fixed at about 30 m^3^/h in the process of experiment. The experiment was carried out from November 19, 2017 to April 6, 2018^24,25^. At the same time, the above equations are also used to simulate the performance of DBHE, and the simulation and experimental results are shown in Fig. 3. In Fig. 3, the symbol *P* represents the thermal power extracted from DBHE, and *T*_in_ and *T*_out_ respectively denote the temperature of injection and extraction water. The average thermal power from DBHE during the experiment process is 448.49 kW. According to the data analysis the maximum deviation between simulation and experimental data in Fig. 3 is 11.69% and the average deviation is 2.95%. It is obvious from Fig. 3 that numerical simulation and experimental results are in good agreement, which demonstrates that the mathematical model here is reliable. The DBHE system in Qingdao, China has been in operation for five heating seasons up till today, and the attenuation degree of the thermal power is less than 0.5% each year, indicating the stability of DBHE.

**Table 3:** Parameters of DBHE in Qingdao, China.

Parameter	Value
Ground surface temperature, °C	15.0
Geothermal gradient, °C/km	27.0
Thermal conductivity of insulation tube, W/(m °C)	0.21
Density of rock, kg/m^3^	2800
Specific heat of rock, J/(kg °C)	920
Thermal conductivity of rock, W/(m °C)	3.49
Thermal conductivity of cement, W/(m °C)	0.73
Outer diameter of insulation tube, mm	110
Insulation tube thickness, mm	10
Thickness of cement, mm	19.05

**Figure 4:**
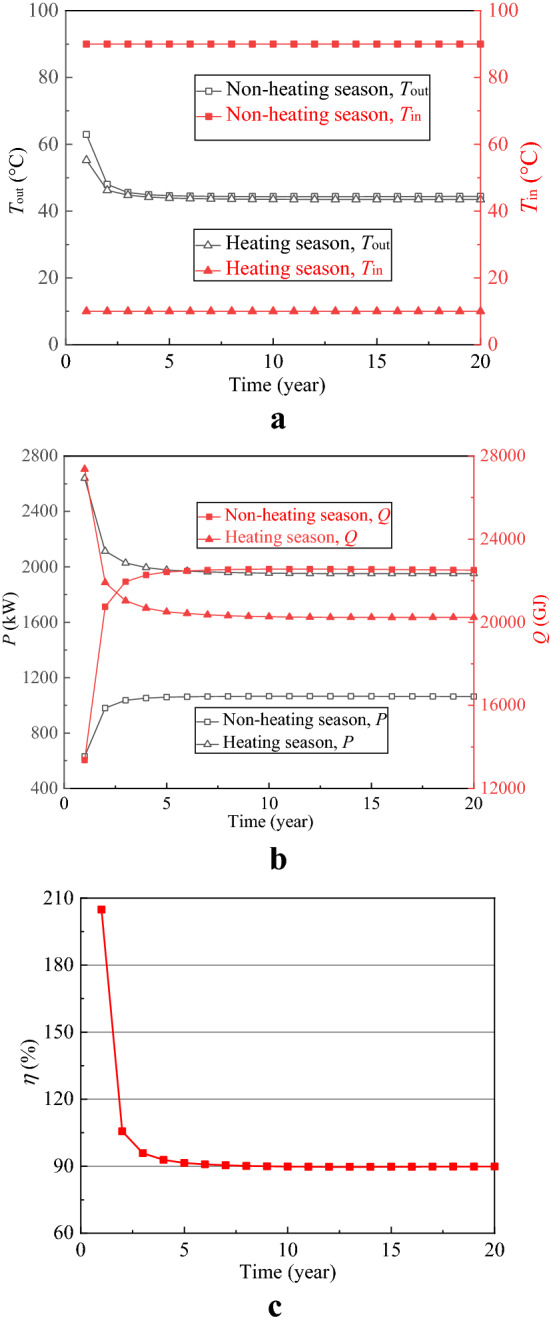
Thermal performance of SDAR. (**a**) Injection and extraction temperature; (**b**) Thermal power and total heat; (**c**) Thermal recovery efficiency.

**Figure 5:**
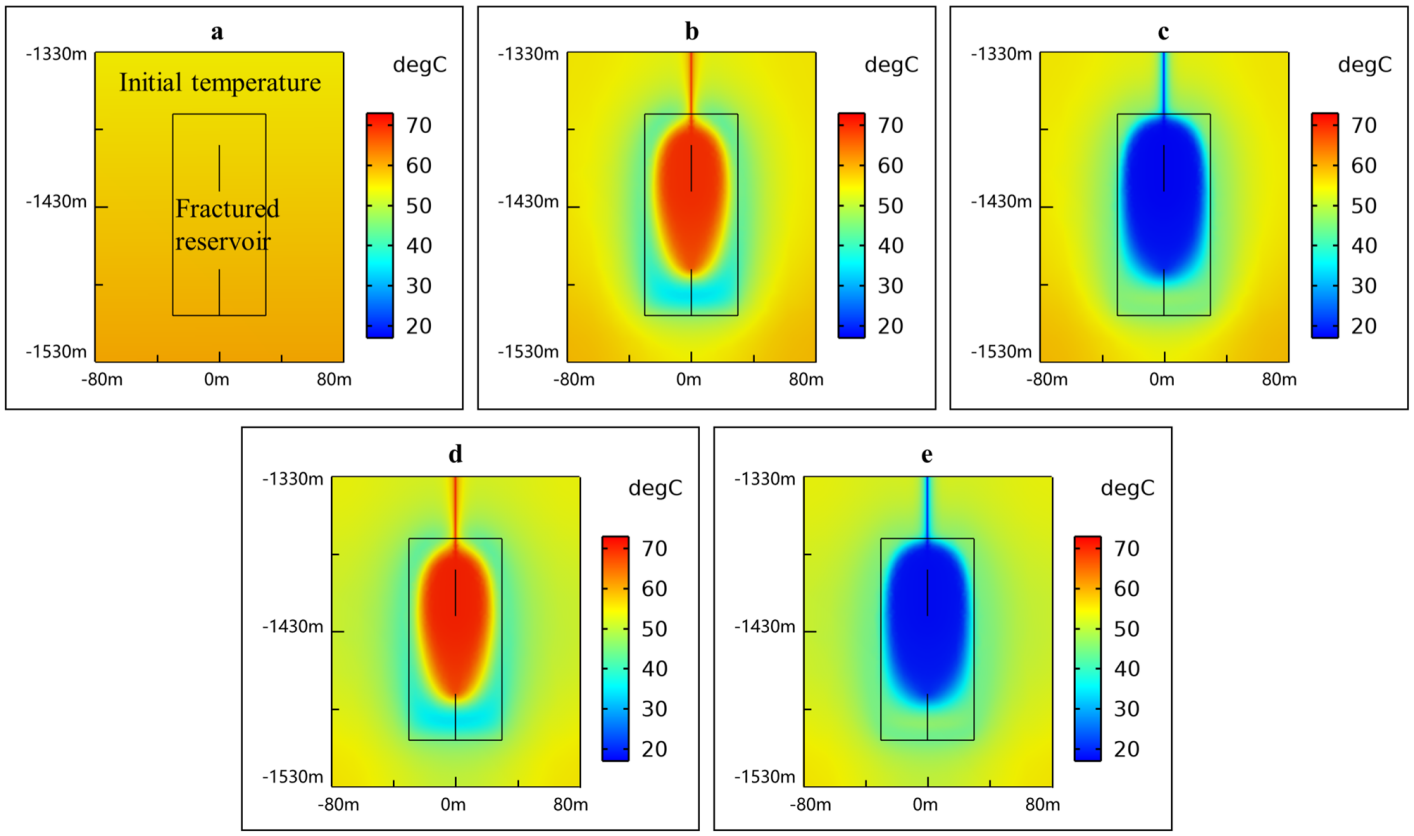
Temperature field distribution around SDAR. (**a**) Initial temperature without thermal energy storage and extraction; (**b**) At the beginning of 10th heating season; (**c**) At the end of 10th heating season; (**d**) At the beginning of 20th heating season; (**e**) At the end of 20th heating season.

The experiment of HTSTESSDAR has not been implemented up till now. To demonstrate the validation of mathematical model for HTSTESSDAR, the thermal energy stored or extracted is analyzed and compared from two perspectives of rocks and working fluid. In term of rocks, the total volume of the artificial reservoir with a height of 130 m and a radius of 30 m is 3.68 × 10^5^ m^3^, and it will release or store 751.49 GJ heat when its temperature changes 1 °C. When the system runs stably, the average total stored or extracted heat each year are respectively 22,390.66 and 20,427.70 GJ (as shown in Fig. 4b) calculated according to the temperature difference between inlet and outlet and the volume flow rate, indicating that the variation of the average rock temperature in the artificial reservoir from the non-heating season to the heating season or from the heating season to the non-heating season should be between 27 and 30 °C when the thermal energy is stored into rocks or extracted from rocks. Observing Fig. 5, the average rock temperatures in the artificial reservoir at the beginning of 10th and 20th heating season are all about 60 °C, and they are all about 30 °C at the end of 10th and 20th heating season, meaning that the temperature difference of rocks in the artificial reservoir is around 30 °C. The above analysis shows that the total heat stored or extracted from the perspective of rocks agrees well with that from the perspective of working fluid. The key is to create an artificial reservoir with the developed and expected fractures so that the thermal energy can be more effectively stored and extracted in the artificial reservoir.

## Results

An artificial reservoir with a height of 130 m and a radius of 30 m is created at the depth from 1370 to 1500 m. During the non-heating season with a duration of 245 days, the high temperature thermal energy produced by the evacuated tubular solar collector is stored into the artificial reservoir with an injection temperature of 90 °C and a volume flow rate of 20 m^3^/h, while during the heating season with a duration of 120 days, it is extracted for space heating with an injection temperature of 10 °C and a volume flow rate of 50 m^3^/h.

In Fig. 4, the symbols *T*_in_ and *T*_out_ stand for the temperature of injection and extraction water, respectively; *P*, *Q* and *η* respectively denote the thermal power, total heat and thermal recovery efficiency. It is apparent from Fig. 4 that *T*_in_, *T*_out_, *P*, *Q* and *η* all keep stable for 20 years’ operation except the first year. Compared Fig. 5a with Fig. 5c,e, it is obvious that the temperature of rocks in the artificial reservoir before the first non-heating season (initial temperature) is higher than that at the end of 10 and 20 heating season, which causes the thermal energy stored in the artificial reservoir to decrease compared with the other non-heating seasons at the same injection temperature and volume flow rate, thus leading to a higher *T*_out_ and a lower *P* and *Q* in the first non-heating season. In Fig. 4b, the average thermal power during the heating season at the stable phase from 2nd year to 20th year is 1970 kW, which can provide heat for 65,667 m^2^ buildings with the specific heat load of 30 W/m^2^. The thermal recovery efficiency *η* is equal to *Q* during the heating season over *Q* during the non-heating season. As shown in Fig. 4c, the thermal recovery efficiency changes little with time from 2nd year to 20th year and keeps stable at 90%. HT-ATES systems typically have lower thermal recovery efficiency due to the thermal advection induced by density contrasts, and the thermal recovery efficiency of HT-ATES created in Neubrandenburg is 46% throughout 3 years of regular operation^26^. Compared with HT-ATES, HTSTESSDAR revolutionizes the way and medium of thermal energy storage and stores thermal energy innovatively into rocks by heat conduction, which circumvents the effects of free convection caused by density difference and thus improves the thermal recovery efficiency. As might be expected, the above analysis shows that storing thermal energy into rocks can improve the thermal recovery efficiency greatly compared with HT-ATES (*η* = 90% for SDAR and *η* = 46% for HT-ATES). Moreover, the integrated analysis of Figs. 4 and 5 indicates that the system of HTSTESSDAR runs stable and has no performance degradation for 20 years’ operation, implying that the performance of HTSTESSDAR only relies on the storage and extraction of thermal energy not on the temperature of rocks, buried depth and water storage space of the artificial reservoir. The point is to highlight that HTSTESSDAR can always provide buildings with a trustworthy and stable thermal energy throughout its life cycle.

**Figure 6:**
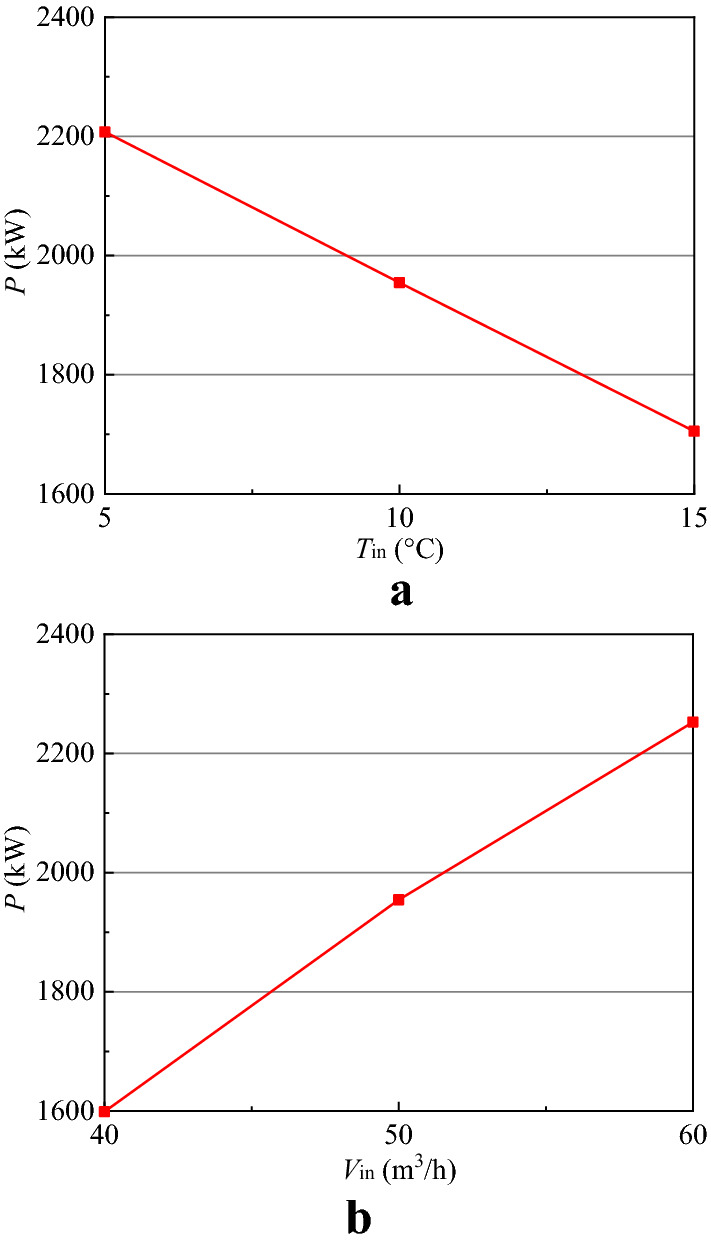
Variations of thermal power of SDAR with injection temperature and volume flow rate. (**a**) Changes in injection temperature with a volume flow rate of 50 m^3^/h; (**b**) Changes in volume flow rate with an injection temperature of 10 °C.

According to Eq. (15) and energy conservation, the evacuated tubular solar collectors with area of 8000 m^2^ are installed. Due to the storage tank storing the short-term heat energy on a diurnal basis, the daily average thermal power of solar energy over 24 h during the non-heating season is 1067 kW, and it is 520 kW during the heating season. During the heating season, the artificial reservoir system and solar energy system are combined to provide heat for buildings. Due to the instability of solar energy, the artificial reservoir should thus have a higher ability of thermal load regulation in order to keep the heating system to run stable during the heating season. Figure 6 shows that the thermal power of the artificial reservoir during the heating season (take 10th heating season as an example) are respectively 2207.61, 1954.49 and 1705.16 kW for the injection temperature of 5, 10 and 15 °C at a constant volume flow rate of 50 m^3^/h, and they are respectively 1598.85, 1954.49 and 2252.58 kW for the volume flow rate of 40, 50 and 60 m^3^/h at a constant injection temperature of 10 °C. That is to say, the thermal power of the artificial reservoir can be adjusted by changing the injection temperature and volume flow rate. Further, the thermal power of the artificial reservoir during the heating season reaches 2553.33 kW at an injection temperature of 5 °C and a volume flow rate of 60 m^3^/h with a thermal load regulation ability of 583.33 kW compared with the average thermal power of 1970 kW obtained at an injection temperature of 10 °C and a volume flow rate of 50 m^3^/h. The heating system can be designed according to the maximum heating load of 2490 kW (1970 kW from the artificial reservoir and 520 kW from solar energy), and the instability of the hybrid heating system (artificial reservoir and solar energy) can be solved by adjusting the thermal power of the artificial reservoir even if solar energy system does not run. One of the fundamental design principles for the hybrid heating system is that the thermal load regulation ability of SDAR should not be less than the daily average thermal power of solar energy in the heating season. The above analysis results also show that two significant benefits can be achieved from the hybrid heating system ① the discontinuous and unstable energy supply problem for solar energy can be solved by storing thermal energy into the artificial reservoir; ② the thermal output of the hybrid system can keep stable by adjusting the thermal power of the artificial reservoir.

**Figure 7:**
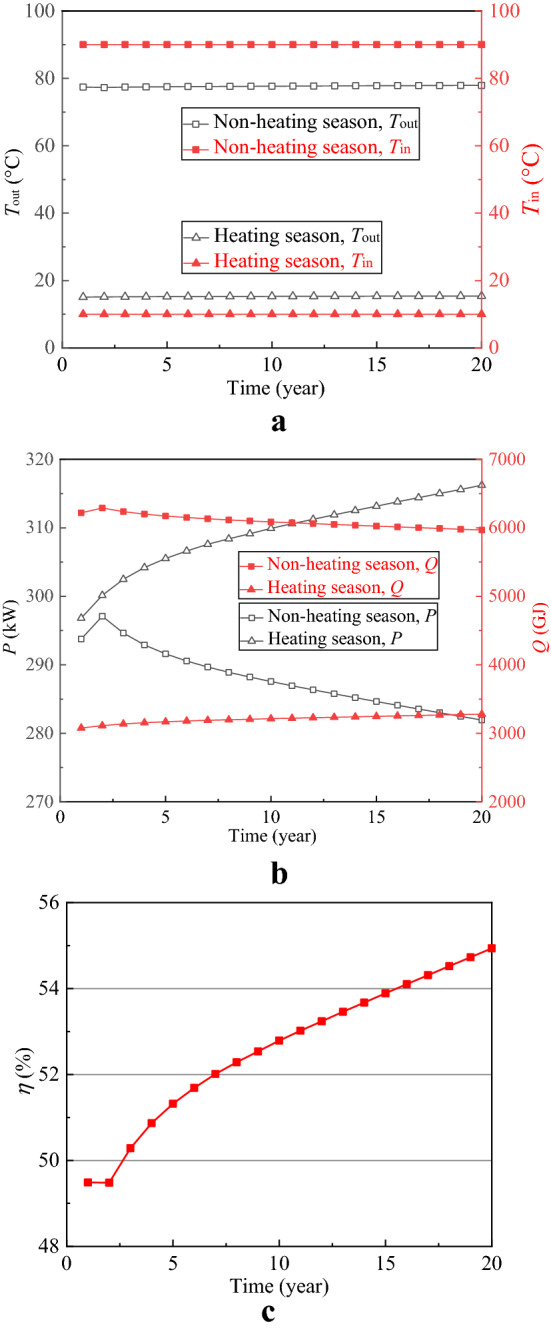
Thermal performance of DBHE. (**a**) Injection and extraction temperature; (**b**) Thermal power and total heat; (**c**) Thermal recovery efficiency.

**Figure 8:**
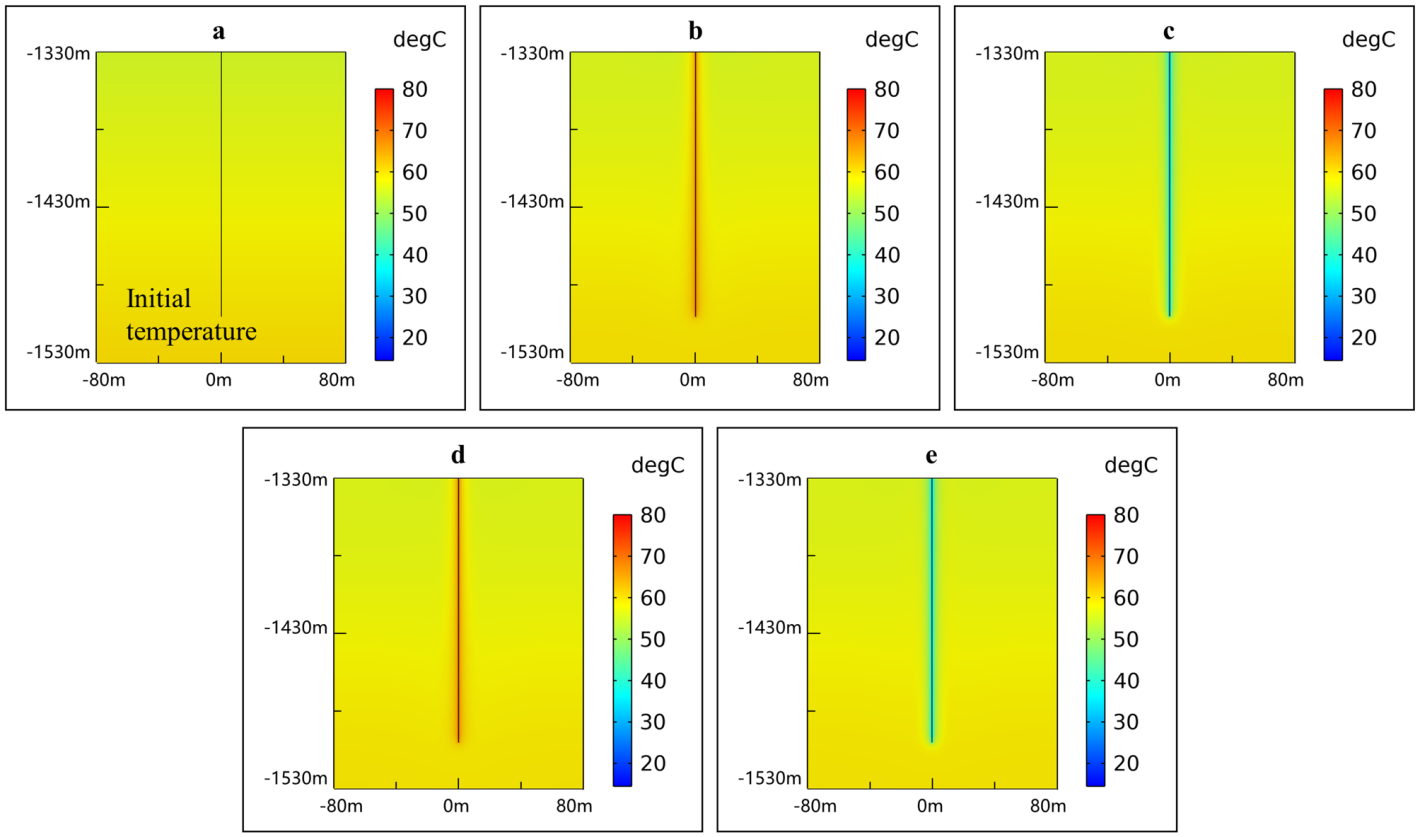
Temperature field distribution around DBHE. (**a**) Initial temperature without thermal energy storage and extraction; (**b**) At the beginning of 10th heating season; (**c**) At the end of 10th heating season; (**d**) At the beginning of 20th heating season; (**e**) At the end of 20th heating season.

Deep borehole heat exchanger (DBHE) (Fig. 2) is a closed loop system and is independent of the hydrothermal resources as well as effectively avoids the problems of reinjection, corrosion and scaling, and thus lots of such projects have been implemented around the world especially in China^27–31^. To compare the performance between SDAR and DBHE, a numerical simulation on DBHE is carried out and the calculation results are shown in Figs. 7 and 8. All parameters for SDAR and DBHE (Tables 1 and 2) in the process of simulation are the same except the artificial reservoir. Figure 7a,b show that *T*_in_ and *T*_out_ remain stable for 20 years’ operation, and the thermal power in the heating season increases with time and however, the percentage increase of the thermal power is only 6.54% (296.81 kW for 1st year, 316.21 kW for 20th year). Figure 7c indicates that the thermal recovery efficiency of DBHE is all low than 55% for 20 years’ operation. Observing the temperature field distribution around DBHE (Fig. 8), it is clear that the temperature of rocks only around the geothermal well changes, while it remains almost unchanged when the rocks are 5 m from the geothermal well. There are two main reasons for the above result. One is the poor thermal conductivity of rocks causing a big thermal resistance between the rocks and the geothermal well; the other is the small heat exchange area between the working fluid and the rocks. As a result, the thermal energy stored in the rocks is hard to transfer to the geothermal well due to the big thermal resistance. It is like a treasure which is located at the top of the hill and however, there is no road to the top.

Compared with DBHE, the artificial reservoir creates a lot of fissures in the rocks and thus increases the heat exchange area as well as reduces the thermal resistance between the rocks and the working fluid, leading to an increase in the thermal power from 309 kW (DBHE) to1970 kW (SDAR). From the point of view of heat transfer, two main factors contribute to the increase in the thermal power of SDAR ① both heat transfer coefficient and heat exchange area all increase in the artificial reservoir compared with DBHE. The coefficient of convection heat transfer between fluid and the rocks in SDAR is far greater than that of thermal conductivity of rocks in DBHE, and the heat exchange area between fluid and the rocks in SDAR having a lot of fissures is also far greater than that of between fluid and the well tube in DBHE; and ② the thermal resistance from the surrounding rocks to the artificial reservoir in SDAR is far less than that from the surrounding rocks to the geothermal well in DBHE due to the diameter of the artificial reservoir in SDAR is far greater than that of geothermal well in DBHE, and thus more heat transfers from the surrounding rocks to the artificial reservoir during the heating season and vice versa. In the case of the thermal conductivity of rocks and heat transfer temperature difference between the fluid and the rocks as well as the injection volume flow rate unchanged, increasing the contact area between the fluid and the rocks is the most effective measure to enhance the heat exchange. The increase of the contact area by creating the artificial reservoir in the shallow depth rocks (SDEGS) is technically easy and low-cost compared with that in the deep EGS. To reduce costs and risks further, the abandoned oil and gas fields and mines can be used to create the artificial reservoir for the storage of high temperature thermal energy.

## Discussion and conclusions

To improve the thermal recovery efficiency, a novel scheme of storing high temperature thermal energy into an artificial reservoir created in the shallow depth rocks is proposed in this study, and it is expected that the thermal recovery efficiency can be greatly improved by storing thermal energy in the rocks instead of aquifer. As expected, the results suggested the thermal recovery efficiency for HTSTESSDAR reaches 90% while it is only 46% for HT-ATES in Neubrandenburg, and the thermal power for HTSTESSDAR increases to 1970 kW while it is only 309 kW for DBHE at the same condition.

For HTSTESSDAR, the artificial reservoir has no special requirement to the rock temperature, and it can thus be created in the shallow buried depth rocks, leading not only to a considerable reduction of project cost but also a great expansion in the application scope. In our opinion, HTSTESSDAR is the last piece of geothermal heating puzzle. Based on the above analysis, we give the most appropriate technical route for geothermal heating ① using LT-ATES in the regions with rich shallow groundwater or using shallow ground source heat pump; ② using geothermal heating or HT-ATES in the regions with rich hydrothermal geothermal resources or deep groundwater and ③ using HTSTESSDAR in the other regions.

## Data Availability

The datasets used and/or analysed during the current study available from the corresponding author on reasonable request.

## List of symbols

*A*_c_: Cross-sectional area of the tube, m^2^
*C*_f_: Specific heat of water, J/(kg K)
*C*_s_: Specific heat of rock, J/(kg K)
*D*: Hydraulic diameter, m
*f*: Darcy friction coefficient
*G*: Direct radiation intensity, W/m^2^
*g*: Gravitational acceleration, m/s^2^
*h*: Convective heat transfer coefficient, W/(m^2^ K)
*h*_II_: Convective heat transfer coefficient for insulation tube inner wall, W/(m^2^ K)
*h*_IO_: Convective heat transfer coefficient for insulation tube outer wall, W/(m^2^ K)
*h*_WI_: Convective heat transfer coefficient for well tube inner wall, W/(m^2^ K)
*k*: Permeability of the artificial reservoir, m^2^
*Nu*: Nusselt number
*p*: Pressure, Pa
*Pr*: Prandtl number
*Q*: Heat transfer item, W/m
*Q*_I_: Heat transfer item for insulation tube, W/m
*Q*_W_: Heat transfer item for well tube, W/m
*q*: Darcy velocity, m/s
*Re*: Reynolds number
*R*_I_: Thermal resistance of insulation tube, (m K)/W
*R*_W_: Thermal resistance of the well tube, (m K)/W
*r*_II_: Insulation tube inner radius, m
*r*_IO_: Insulation tube outer radius, m
*r*_WI_: Well tube inner radius, m
*r*_WO_: Well tube outer radius, m
*T*: Temperature, K
*TE*: Thermal efficiency
*T*_E_: Extraction fluid temperature, K
*T*_I_: Injection fluid temperature, K
*T*_m_: Average outlet and inlet temperature of solar collector, °C
*T*_A_: Ambient temperature, °C
*T*_s_: Rock temperature, K
*t*: Time, s
*u*_f_: Flow rate of water, m/s
*ρ*_f_: Water density, kg/m^3^
*ρ*_s_: Rock density, kg/m^3^
*λ*_e_: Effective thermal conductivity of artificial reservoir, W/(m K)
*λ*_f_: Thermal conductivity of water, W/(m K)
*λ*_I_: Insulation tube thermal conductivity, W/(m K)
*λ*_W_: Well tube thermal conductivity, W/(m K)
*λ*_s_: Rock thermal conductivity, W/(m K)
*ϕ*: Porosity of the artificial reservoir
*μ*_f_: Fluid viscosity, Pa s
